# The impact of a musculoskeletal training program on residents’ recognition and treatment of osteoporosis

**DOI:** 10.1186/s12909-019-1653-4

**Published:** 2019-06-21

**Authors:** Richard E. Nelson, Junjie Ma, Karla Miller, Phillip Lawrence, Joanne LaFleur, Marissa Grotzke, Andrea Barker, Grant W. Cannon, Michael J. Battistone

**Affiliations:** 1 IDEAS Center, Veterans Affairs Salt Lake City Health Care System, 500 Foothill Blvd, Salt Lake City, UT 84148 USA; 2 University of Utah School of Medicine, Salt Lake City, UT USA; 3 University of Utah College of Pharmacy, Salt Lake City, UT USA

**Keywords:** Osteoporosis, Provider training, Veterans

## Abstract

**Background:**

Osteoporosis is inadequately treated in primary care settings. Under-recognition of the condition among male Veterans may contribute to this problem. In order to improve understanding of bone health in older male patients, we developed the “Musculoskeletal (MSK) Education Week”, a multidisciplinary clinical training initiative within a primary care ambulatory rotation for internal medicine (IM) residents at the Salt Lake City VA Medical Center. The objective of this study was to evaluate the impact of this program on trainees’ recognition of osteoporosis or treatment of this condition following the training experience.

**Methods:**

We examined several clinical behaviors of post-graduate year 1 (PGY-1) IM trainees following their participation in the MSK Education Week between July 1–April 30, 2014. To determine the prevalence of these clinical behaviors, we conducted an observational study of patients age 50 and older enrolled at the Salt Lake City VA Healthcare System from July 1, 2013 to May 31, 2014. We used time-dependent multivariable Cox proportional hazard models to evaluate the impact of the training program on 4 osteoporosis-related outcomes: (1) completion of dual energy X-ray absorptiometry (DXA) scan, (2) diagnosis of osteopenia, (3) diagnosis of osteoporosis, and (4) initiation of osteoporosis medications.

**Results:**

Twenty-six PGY-1 IM residents participated in the MSK Education Week, and 43,678 Veterans were identified over these periods of observation. In the Veterans cohort, 1154 had an encounter with a provider who had completed the training (and were therefore “exposed” to the training) and 42,524 Veterans did not. After adjusting for confounders, the effect of the provider training program was significant for DXA (HR = 1.78, 95% CI: 1.11, 2.87), osteoporosis diagnosis (HR = 3.90, 95% CI: 2.09, 7.29), and initiation of medications (HR = 2.87, 95% CI: 2.02, 4.09) outcomes.

**Conclusions:**

We have shown that IM residents’ participation in the MSK Education Week was associated with significantly improvements in their completion of DXA scans, diagnosis of osteoporosis, and initiation of fracture-reducing medications in a population of US Veterans. Long-term follow up is needed to determine whether these initial results are followed by actual reductions in osteoporotic fractures.

## Background

More than 10 million adults in the United States age 50 or older have osteoporosis, leading to a prevalence of 10.3% in this age group. Moreover, when including adults with osteopenia, the prevalence of overall low bone mass is 43.9% in older adults [1]. It is expected that, by 2025, the total cost of osteoporosis-related fractures in the United States will be more than $25 billion [2]. Although low bone mass is common, bone density screening is underutilized, especially in older men. According to a systematic review, the screening frequencies of at-risk population ranged from 1 to 47% [3]. In addition, a large scale observational study found that nearly two-thirds of patients did not receive osteoporosis medications during 1-year period following an osteoporosis diagnosis [4]. Similar issues are seen within the Department of Veterans Affairs (VA) where less than one-quarter of Veterans who experience a low-trauma fracture are appropriately evaluated and treated [5].

One possible solution to increase osteoporosis awareness among clinicians is through provider education programs. Such programs have been shown to improve clinician knowledge and clinical practice in other clinical areas. For example, a recent randomized trial of a physician education program for asthma designed to improve therapeutic and communication skills found that the patients whose primary care physician had attended the training program had fewer symptomatic days and fewer asthma-related emergency department visits [6]. However, there have been relatively few published descriptions of structured educational programs for health care providers or trainees in the area of musculoskeletal (MSK) care.

In 2012, we established the Center of Excellence (COE) in patient-centered MSK Care and Education, a key component of which is the monthly “MSK Education Week”, a multidisciplinary clinical training initiative developed within a primary care ambulatory rotation which is now required for all post-graduate year 1 (PGY-1) internal medicine (IM) residents [7]. This structured educational experience cultivates both confidence and competence; learners from a wide range of training backgrounds and levels report increased ability in performing shoulder and knee exams, and are able to demonstrate these skills in a two-station observed structured clinical examination (OSCE) [8]. The scope of the COE MSK Education Week includes training in metabolic bone disease. Risk factors, diagnostic definitions, guidelines for screening, and current treatment options for osteopenia and osteoporosis are discussed by an endocrinologist with expertise in bone health.

The objective of this study was to evaluate the impact of the COE MSK Education Week on IM residents’ osteoporosis management behaviors in their subsequent ambulatory care training experiences. We did this by assessing outcomes capturing osteoporosis treatment or recognition following participation in this training.

## Methods

### COE MSK week program and trainee cohort

The development, implementation, and three-year sustainment of the COE MSK Education Week have been previously described [7]. This five-day, structured, immersive educational experience is held each month, and attended on average by 6–8 participants who are health professions students or postgraduate trainees. Over the five days of this course, two half-days are focused on metabolic aspects of bone health, which is organized across in five modules: 1) osteoporosis definitions, screening guidelines, and diagnostic pathways; 2) the use of fracture risk assessment tools, including FRAX; 3) the evaluation, prevention, and treatment of vitamin D deficiency; 4) osteoporosis treatment options; and 5) the clinical assessment of treatment outcomes, with particular emphasis on when to refer patients for subspecialty care. A printed syllabus containing information relevant to these modules is provided at the beginning of the week, and is reviewed in the half-day sessions in a structured, interactive discussion format facilitated by an endocrinologist with expertise in osteoporosis (MG). Supervised clinical experiences later in the week reinforce this learning by providing opportunities to use this information in patient care.

The trainee cohort consisted of 26 PGY1 IM categorical residents who participated in the MSK Education Week at some point during July 12,013–April 30, 2014. Of these 26 MSK Education Week participants, 8 (31%) were female and the mean (SD) age at the time of training was 29.4 (4.2). Although the VHASLC serves as a clinical training affiliate for other health professions education programs, including “preliminary” PGY-1 residents preparing for specialty training (e.g., anesthesia, neurology, etc.), these learners did not participate in the MSK Education Week and were not included in the definition of the trainee cohort.

### Study design and patient cohort

We conducted a historical cohort study of patients age 50 and older enrolled at the SLCVAMC for primary care. To ensure that those patients in the study were regular users of the VA healthcare system, we excluded patients who did not have a primary care encounter within 6 months prior to index date (July 1, 2013). The study was reviewed by the University of Utah Institutional Review Board and determined an observation report of quality improvement data to be exempt from Human Studies Review.

### Exposures and outcomes

The exposure for our analysis was a treatment episode with a provider who had completed the COE MSK Education Week. This was a time-varying exposure as patients in our study encountered COE-trained providers based on the timing of their clinical appointments and the timing of the providers’ COE training. The outcomes in this study included (1) completion of dual energy X-ray absorptiometry (DXA) scan (2), diagnosis of osteopenia (3), diagnosis of osteoporosis, and (4) initiation of osteoporosis medications.

### Analysis

Patient characteristics were compared between patients who were treated by a COE-trained provider and those who were not using chi-square tests. We calculated crude rates per 10,000 patient-days and rate ratios for each outcome for both groups. The 95% confidence intervals for these rates were constructed using a method that relates the chi-square and Poisson distributions [9]. In addition, these rates were used to calculate unadjusted rate ratios between the two groups. Finally, we used time-dependent, multivariable Cox proportional hazards models to evaluate the impact of the COE program on these outcomes. This analysis evaluated the impact of the first year of the COE MSK Education Week trainings. In our analysis, patients were followed – with time characterized by days – beginning on the index date until either having an outcome event or being censored on May 31, 2014. Patients contributed person-time to the non-COE group prior to being exposed to a COE-trained provider. A Cox proportional hazards model was chosen for this analysis due to the ability of this approach to efficiently handle a time-varying exposure.

Our multivariable models adjusted for potential confounders in the relationship between COE MSK Education Week and osteoporosis management outcomes such as age, sex, alcohol use, tobacco use, diabetes, prior fractures, hyperparathyroidism, vitamin D deficiency, renal disease, and medication exposures in our multivariable models. The diagnoses were identified using ICD-9 codes and medication exposures included anticonvulsants, aromatase inhibitors, androgen deprivation therapy, and testosterone.

### Data

This study was conducted using data from the Corporate Data Warehouse (CDW), which is a national repository of data from the VA electronic medical record and several other VA clinical and administrative systems. Through the CDW, we identified diagnoses through International Classification of Diseases, Ninth Revision, Clinical Modification (ICD-9) codes. These codes included 733.00, 733.01, 733.02, 33.03, and 733.09 for osteoporosis and 733.90 for osteopenia. Pharmacy records were used to identify osteoporosis medications dispensed and included alendronate, risidronate, ibandronate, calcitonin, raloxifene, teriperatide, and denosumab. Using these datasets, we constructed indicator variables for each of these outcomes if the item was present.

## Results

Table 1 depicts the characteristics of the 43,678 Veterans in our analysis cohorts. Of the patients in our cohort, 1154 had an encounter with a provider who had completed the COE MSK Education Week (and were therefore “exposed” to the training) and 42,524 patients did not. The patients in the MSK cohort were significantly older compared to the patients in the non-MSK cohort (*p* < 0.0001). Across both groups, a majority of patients were white (82.0%), although the proportion of whites was significantly higher in the MSK group (81.7% vs. 91.2%, *p* < 0.0001). Patients in the MSK group were more likely to be male (94.2% vs. 91.3%, *p* < 0.0001) but the overwhelming majority in both groups were male. Finally, patients in the MSK cohort were more likely to have previous exposure to steroids and testosterone (12.2% vs. 2.9%, *p* < 0.001; 3.6% vs. 1.7%, *p* < 0.0001).

**Table 1:** Patient characteristics for study cohort

	COE		Non-COE		
	N	%	N	%	*p*-value
Demographics^a^					
Age					
< 65	533	46.2%	23,390	55.0%	
65–74	324	28.1%	10,369	24.4%	< 0.0001
75–80	86	7.5%	2679	6.3%	
80–85	94	8.1%	2842	6.7%	
85+	117	10.1%	3244	7.6%	
Male	1087	94.2%	38,823	91.3%	0.001
White	1053	91.2%	34,752	81.7%	< 0.0001
Medication use					
Estrogen	2	0.2%	38	0.1%	0.285
Hormone deprivation meds	7	0.6%	93	0.2%	0.017
Seizure meds	1	0.1%	45	0.1%	0.656
Steroid	141	12.2%	1244	2.9%	< 0.0001
Testosterone	42	3.6%	718	1.7%	< 0.0001
Comorbidities					
Alcohol	7	0.6%	113	0.3%	0.040
Diabetes	47	4.1%	513	1.2%	< 0.0001
Fall	34	2.9%	370	0.9%	< 0.0001
Hyperparathyroidism	8	0.7%	83	0.2%	0.003
Malnutrition	56	4.9%	598	1.4%	< 0.0001
Prior fracture	6	0.5%	161	0.4%	0.460
Renal disease	108	9.4%	1027	2.4%	< 0.0001
Rheumatoid arthritis	16	1.4%	203	0.5%	< 0.0001
Smoking	207	17.9%	4196	9.9%	< 0.0001
Stroke	30	2.6%	462	1.1%	< 0.0001
Vitamin D deficiency	23	2.0%	505	1.2%	0.019

*Note:* ^a^As of index date (July 1, 2013)

Unadjusted rates for each of the outcomes, along with 95% confidence intervals (CIs), are presented in Table 2. Patients with an encounter with a provider who had participated in the COE MSK Education Week were more likely to have each outcome when compared to patients in the non-MSK cohort, with the strongest effect being seen in the diagnosis of osteoporosis (RR = 7.09, 95% CI: 4.41, 11.21) and completion of DXA scan (RR = 4.36, 95% CI: 3.02, 6.06).

**Table 2:** - Osteoporosis surrogate outcomes – crude rates

	COE Patients					Non-COE Patients					Rate Ratio		
				95% CI					95% CI			95% CI	
Outcome	Number of patients with event	Person-time (days)	Rate^a^	LL	UL	Number of patients with event	Person-time (days)	Rate^a^	LL	UL	Rate Ratio	LL	UL
Osteoporosis treatment	5	160,027	0.312	0.101	0.729	107	14,026,193	0.076	0.063	0.092	4.10	1.79	9.36
DXA	28	155,527	1.800	1.196	2.602	576	13,956,364	0.413	0.380	0.448	4.36	3.02	6.06
Osteopenia diagnosis	7	159,872	0.438	0.176	0.902	266	14,005,290	0.190	0.168	0.214	2.31	1.11	4.77
Osteoporosis diagnosis	14	159,052	0.880	0.481	1.477	174	14,014,687	0.124	0.106	0.144	7.09	4.41	11.21

^a^per 10,000 patient-days

*Note:* CI = confidence interval, *LL* = lower limit, *UL* = upper limit, *COE* = Center of Excellence, *DXA* = dual energy X-ray absorptiometry

Results from the univariate and multivariable regression models are summarized in Table 3. In the univariate analyses, participation in the COE MSK Education Week was associated with an increase in each of the outcomes, with the HRs (95% CI) ranging from 6.96 (3.77–12.82) for osteoporosis diagnosis to 2.41 (1.52–3.85) for osteopenia diagnosis. After adjusting for confounders, the MSK training was still associated with an increase of the outcomes measured and was statistically significant for DXA scan (HR = 1.78, 95% CI: 1.11, 2.87), osteoporosis diagnosis (HR = 3.90, 95% CI: 2.09, 7.29), and treatment initiation (HR = 2.87, 95% CI: 2.02, 4.09) outcomes.

**Table 3:** – Results from univariate and multivariate Cox proportional hazards regression

	Univariate Results				Multivariable Results			
		95% CI				95% CI		
Outcome	HR	LL	UL	*P*-value	HR	LL	UL	*P*-value
Osteoporosis treatment	3.403	2.400	4.825	<.0001	2.874	2.018	4.093	<.0001
DXA	3.300	2.063	5.279	<.0001	1.782	1.109	2.865	0.017
Osteopenia diagnosis	2.414	1.515	3.846	<.0001	1.324	0.829	2.115	0.240
Osteoporosis diagnosis	6.955	3.773	12.821	<.0001	3.904	2.090	7.293	<.0001

*Note: HR* = hazard ratio, *CI* = confidence interval, *LL* = lower limit, *UL* = upper limit, *DXA* = dual energy X-ray absorptiometry. Multivariable regression models controlled for age, sex, alcohol use, tobacco use, diabetes, prior fractures, hyperparathyroidism, vitamin D deficiency, renal disease, and medication exposures

## Discussion

In this paper, we examined an innovative education program designed to increase the rate at which medical residents utilize screening and treatment for patients at risk for osteoporosis. We explored the association between the implementation of this program and a number of intermediate MSK outcomes. The ideal outcome to assess in these analyses would have been fragility fractures. However, because they are relatively rare events, fractures were not well-suited for an analysis within a short time frame. The intermediate outcomes that we assessed are highly correlated with future fracture risk so it is possible that an effect on fracture may be seen in the future. After controlling for confounders through a multivariable regression model, we found that treatment with bisphosphonate medications, completion of DXA scan, and diagnosis of osteoporosis in patients from the COE-MSK Week group were significantly improved when compared to patients from the non-COE MSK Week cohort.

We observed that the COE-MSK Week cohort has higher rates of co-morbid conditions and medications use in comparison the non-COE-MSK Week group. This difference may in part be due to the tendency to assign patients with more complex medical conditions to trainee clinics at academic institutions. Our multivariable analysis was designed to address this issue; however, the potential for residual confounding cannot be fully excluded.

Our results can be placed in the context of those from other published studies. For example, a randomized controlled trial including 828 primary care providers (PCPs) and 13,455 patients evaluated the impact of an education program on undergoing a bone mineral density (BMD) test and initiating a fracture reducing medication. The study found that the brief program of patient and physician education did not improve osteoporosis management [10]. An observational study found that a PCP workshop was associated with higher rates of BMD testing and initiation with bone-specific medications in elderly women, but did not significantly improve osteoporosis management in men [11]. Our study showed that the COE MSK Education Week could improve the completion of DXA and diagnosis of osteoporosis. The difference between the current study and previous studies may be due to differences in the design of the education programs with greater training intensity during the MSK Week.

A number of randomized controlled trials have examined interventions to improve management of patients at risk for osteoporosis following a fracture event. Examples of these interventions include patient education through a PCP [12, 13], an electronic medical record reminder to a PCP or a patient [14], education for both the PCP and the patient [15–17], and strategies to shift the responsibility for diagnosing and treating the osteoporosis from the PCP to another provider [18, 19]. A systematic review found that these interventions yielded a risk ratio for BMD screening of 2.80 (95% CI: 2.16–3.64) and a risk ratio for anti-resorptive therapy of 2.48 (95% CI: 1.92–3.20) [20]. These are similar to the effect sizes that we found in our study.

Finally, our results can be compared with those from a knowledge translation tool involving a risk assessment questionnaire that generates patient-specific treatment recommendations and education at the point of care. Kastner et al. found that this tool was associated with significant increases in DXA screening (3.4%; 95% CI: 2.0–4.7) and treatment with osteoporosis medications (0.5%; 95% CI: 0.2–0.9) for patients at risk for osteoporosis. However, the magnitudes of these increases were much smaller than our findings.

It is important to note that our study has several limitations. Although we controlled for a number of covariates in the regression models, this approach may not be sufficient to eliminate all of the confounding caused by the difference between two groups. There may be important provider-level characteristics that influenced both the enrollment in the COE training program and the outcomes in our study. These may include diagnostic skills, intellectual interest in bone health, or previous bone health training. Unfortunately, these provider-level characteristics were not available in our data. Another limitation is that, because this study was conducted in a VA population, it may be difficult to generalize the findings to other healthcare systems. For instance, our cohort was overwhelmingly male (91.4%). In addition, because we only used VA data in our study, events that occurred outside the VA system were not captured in the variables constructed for our analyses. Finally, although we believe that the increased rates of screening and prescription reflect retention and application of principles learned during the MSK Education Week, it is possible that this knowledge was acquired in other rotations. In that case, however, the effect would be expected across the comparison patient cohort as well, and we would not expect to see the statistical significance of the results we have observed.

Despite these limitations, our study had a number of strengths that contribute to the importance of our findings. First, we utilized the VA’s extensive electronic medical record to capture both exposure and outcomes. Second, while Veterans often receive healthcare in non-VA settings [21], the VA is the largest integrated healthcare system in the US [22]. This, along with having selected patients to be included in our study based on having had a primary care encounter prior to the index date, gives us confidence that we have captured most of the outcomes data for the patients in our cohort. Third, our measurement of objective outcomes complements the self-assessment outcomes that are typically captured in evaluations of healthcare educational programs. And finally, our analysis utilized a large control group, which consisted of all Veterans who had been assigned a primary care provider at the SLCVAMC who did not have an encounter with a provider who had been trained through the COE program.

## Conclusion

The implementation of the COE in musculoskeletal care was associated with significantly increased the completion of DXA scan and diagnosis of osteoporosis in a population of US veterans in the SLCVAMC. However, initiation of fracture-reducing medications, and diagnosis of osteopenia were not significantly different in patients who were seen by providers who had received COE training compared to patients who were seen by providers who had not participated in this training.

## Data Availability

The data used for these analyses is located within VA Informatics and Computing Infrastructure (VINCI), a secure, central analytic platform for performing research and supporting clinical operations activities within the Department of Veterans Affairs (VA). As dictated by VA policies, the data for this study (and any other study conducted within VINCI) cannot be removed from this remote computing environment. However, access to the data can be granted to credentialed researchers.

## Abbreviations

BMD: Bone mineral density
CI: Confidence interval
COE: Center of Excellence
DXA: Dual energy X-ray absorptiometry
IM: Internal medicine
MSK: Musculoskeletal
OSCE: Observed structured clinical examination
PCP: Primary care provider
SLCVAMC: Salt Lake City VA Medical Center
VA: Department of Veterans Affairs
